# Appendiceal duplication with Amyand’s hernia: a case report

**DOI:** 10.1093/jscr/rjag234

**Published:** 2026-04-11

**Authors:** Deyna Camila Ripe, Juan Sebastián Cuellar, Gloria Buitrago Lozano, Martha Milena Alfonso

**Affiliations:** Department of Epidemiology, Juan N. Corpas University Foundation, Calle 74A 116 B 60, Bogota 111031, Colombia; Department of General Surgery, Nueva Granada Military University, Calle 116 15 Bogota 110111, Colombia; Department of General Surgery, Juan N. Corpas University Foundation, Calle 135 92 B 25, Bogota 111161, Colombia; Department of General Surgery, University of Rosario, Calle 146A 58 C 56, Bogota 111611, Colombia

**Keywords:** appendicitis, duplication, hernia, inguinal, Amyand, laparoscopy

## Abstract

Appendiceal pathologies and inguinal hernias are common in general surgery; however, exceptional presentations exist, primarily associated with congenital malformations and anatomical anomalies. Appendiceal duplication and Amyand’s hernia are rare entities that present diagnostic and therapeutic challenges. The first reported case of Amyand’s hernia associated with appendiceal duplication in a young female patient is presented.

## Introduction

Appendiceal duplication (AD) is usually diagnosed intraoperatively and is managed according to the type of presentation. When appendiceal tissue is found within the hernial sac, it is called Amyand’s hernia (AH). In both cases, signs of appendicitis should be sought, prophylactic appendectomy considered, and in the case of AH, the decision to postpone hernial repair should be made, as there is controversy regarding the use of surgical mesh due to the risk of infection.

## Case presentation

A 39-year-old female patient, with no prior medical or surgical history, presented to a secondary care center with a 2-day history of lower abdominal pain that subsequently localized to the right iliac fossa, associated with vomiting and a fever of 38.3°C. She reported no urinary symptoms or leukorrhea. On physical examination, she was febrile, tachycardic, with tenderness to palpation in the lower abdomen, a palpable mass, with signs of local infection in the hypogastrium (redness, warmth, and pain), and involuntary guarding; there was no evident swelling. Laboratory studies showed leukocytosis of 37.700/μl and neutrophilia of 87%. Diagnostic imaging was not performed due to unavailability. Due to the clinical presentation of an acute abdomen with an AIR Score of 11 points, an exploratory laparoscopy was performed [1] (Table 1).

**Table 1:** Appendicitis inflammatory response (AIR) score [1]

**Parameters**	**Score**
Vomiting	1
Pain in the right lower quadrant	1
**Rebound tenderness**	
Light	1
Medium	2
Strong	3
**Polymorphonuclear leukocytes**	
70%–84%	1
≥85%	2
**White blood cell count**	
10 000–14 999 cells/μl	1
≥15 000 cells/μl	2
**C-reactive protein**	
10–49 mg/l	1
≥50 mg/l	2
**Total**	
0–4	Low probability
5–8	Mild probability
9–12	High probability

**Figure 1:**
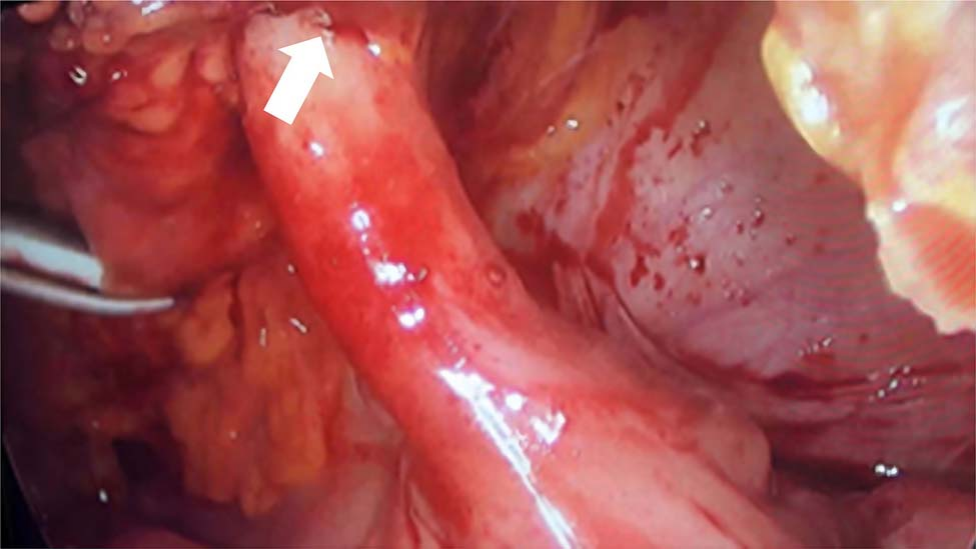
Intraoperative laparoscopic view showing the cecal appendix entering the right inguinal canal (arrow), consistent with an Amyand hernia.

**Figure 2:**
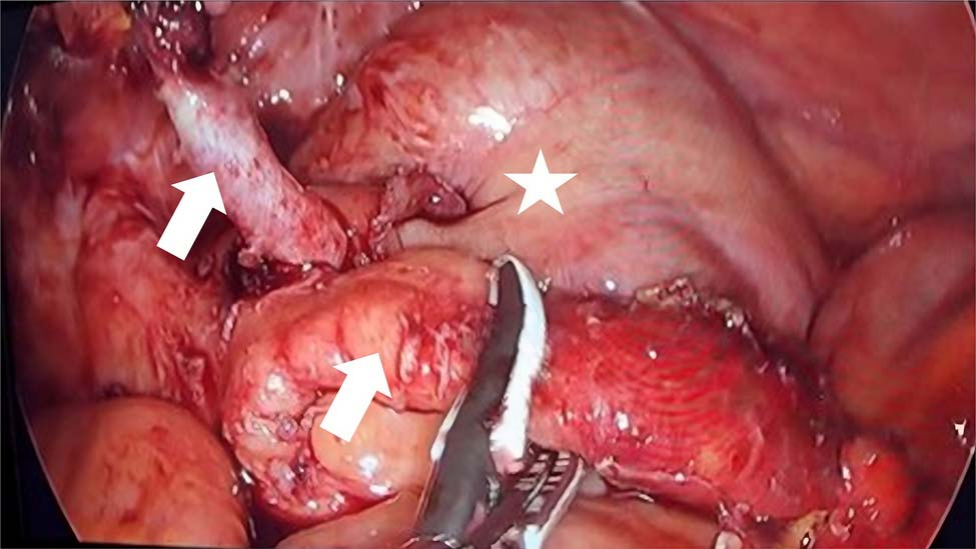
Intraoperative laparoscopic view demonstrating appendiceal duplication with two cecal appendices (arrows), corresponding to Wallbridge Type B1. The arrows indicate both appendices: The left appendix shows perforation, while the right presents inflammatory changes consistent with periappendicitis. The asterisk indicates the cecum with inflammatory reaction.

During the surgical procedure, the cecum was identified with signs of inflammatory reaction, and a tubular structure consistent with the appendix was found within the right inguinal hernia (Fig. 1). Upon release of the hernial sac and dissection of the preperitoneal plane, approximately 500 cc of purulent-appearing fluid was drained. Subsequently, a second appendicular structure was observed, one perforated in the middle third and the other showing signs of periappendicitis, findings consistent with a Cave–Wallbridge 1B type appendicular duplication (Fig. 2 and Table 2) [2]. Both appendectomies were performed with surgical stump control using non-absorbable polymer clips.

**Table 2:** Cave–Wallbridge classification of appendiceal duplication [2]

**Type**	**Description**	**Illustration**
**Type A**	Partial duplication of the appendix-like structure with one normal appendix is present at the base.	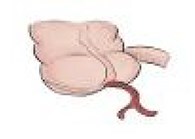
**Type B1**	Due to failure of proper cloacal differentiation, two separate appendices originate from the singular cecum close to ileocecal valve.	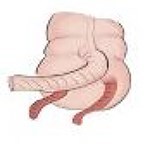
**Type B2**	The duplicated appendix is present along some colon tenia line; both the appendices originate from a single cecum.	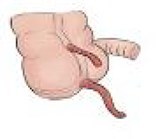
**Type C**	Incomplete duplication of the hindgut results in two separate appendices originating from two separate cecum.	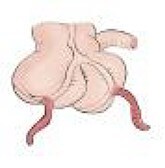
**Type D**	With a common duct, two appendices originate from a single cecum present in a relatively parallel fashion.	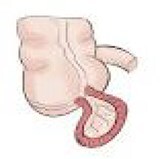

Finally, the preperitoneal space was thoroughly irrigated using sterile gauze wicks with antiseptic solution, and a Blake drain was placed through a counter-incision. Given the degree of contamination of the inguinal surgical field and the risk of contamination of the non-biological mesh, it was decided to postpone the hernia repair. The patient received antibiotic treatment with a carbapenem for 10 days and had a favorable postoperative course. The histopathological report confirmed the presence of two cecal appendices, the first with acute appendicitis and the second with inflammatory changes consistent with periappendicitis.

## Discussion

Appendiceal duplication is an anatomical anomaly with a prevalence of 0.004%–0.009%. Its etiology is related to genetic factors and abnormalities during embryological development [3]. Preoperative diagnosis of AD has been detected using radiological techniques with a barium enema and is confirmed by histopathology when both tissues had lined lumens by appendiceal mucosa, lymphoid follicles, and two layers of muscle. The most widely used classification of AD is the Cave–Wallbridge classification, with category B2 being the most frequent [3–5] (Table 2). Treatment consists of careful resection of both appendices, taking care not to leave any appendiceal remnants [6].

An appendix contained within an inguinal hernia sac is known as an AH, with a prevalence ranging from 0.2% to 2%. Its etiology is related to the migration of the vermiform appendix into the hernia sac through the processus vaginalis [7]. AH can be associated with appendicitis in 0.1% of cases and is related to trauma of the appendiceal base against the neck of the hernia sac, causing increased intra-abdominal pressure. The AH does not usually present with the characteristic symptoms of appendicitis (right iliac fossa pain); instead, it mimics a strangulated hernia with pain in the inguinal region [8, 9].

The diagnosis of AH is not very sensitive. On physical examination, it can be identified as an increase in volume in the inguinal region, along with pain on palpation in the right iliac fossa [10]. It can be observed by computed tomography to identify the appendix entering the inguinal canal; however, it is considered more of an intraoperative finding [8]. Elevated inflammatory markers such as PCR and leukocytosis occur in the presence of appendicitis [11]. In addition, differential diagnoses such as Richter’s hernia, inguinal adenitis, epididymitis, and even testicular torsion should be considered [9].

Regarding surgical management, the modified Losanoff–Basson classification describes the approach for each type of AH. Type 1 AH (appendix without signs of appendicitis) does not benefit from prophylactic appendectomy due to the risk of infection, in addition to studies highlighting the appendix as an organ with immunological functions [12]. Prophylactic appendectomy is chosen for left-sided presentations due to future diagnostic difficulty and in pediatric cases due to the risk of developing appendicitis in the first decades of life [7, 13]. In the remaining presentations, appendectomy is recommended as they are associated with signs of appendicitis (Table 3).

**Table 3:** Modified Rikki classification for Amyand hernia, based on the Losanoff and Basson classification [12]

**Classification**	**Description**	**Surgical management**
**Type 1**	Normal appendix within an inguinal hernia.	Hernia reduction, mesh repair, appendectomy in young patients.
**Type 2**	Acute appendicitis within an inguinal hernia without abdominal sepsis.	Appendectomy for hernia, primary hernia repair without mesh.
**Type 3**	Acute appendicitis within an inguinal hernia, abdominal wall, or peritoneal sepsis.	Laparotomy, appendectomy, primary hernia repair, without mesh.
**Type 4**	Acute appendicitis within an inguinal hernia, related abdominal pathology, or not.	Manage as a type 1 to 3 hernia, investigate, or treat the second pathology as appropriate.
**Type 5a**	Normal appendix within an incisional hernia.	Appendectomy through the wound, primary hernia repair including mesh
**Type 5b**	Acute appendicitis within an incisional hernia without abdominal sepsis.	Appendectomy for hernia, primary wound repair.
**Type 5c**	Acute appendicitis within an incisional hernia, abdominal wall, or peritoneal sepsis or in relation to previous surgery.	Manage as a type 4 hernia.

The gold standard for hernia repair is the use of surgical mesh because it reduces the recurrence rate by up to 75% [14]. However, the use of mesh in contaminated surgical fields carries a higher risk of infection. A systematic review reports that mesh herniorrhaphy was preferred in a second intervention in 42.8% of cases. In contrast, there are reports of AH associated with appendicitis treated with mesh herniorrhaphy, sustained antibiotic therapy, and postoperative drainage without postoperative complications; however, the presence of a perforation abscess was not described [7]. Complications associated with AD and AH include colonic perforation, mechanical bowel obstruction, intra-abdominal abscess, and peritonitis, with a mortality rate of up to 30% [7, 15].
